# Fabrication of Micro-Ball Sockets in C17200 Beryllium Copper Alloy by Micro-Electrical Discharge Machining Milling

**DOI:** 10.3390/ma16010323

**Published:** 2022-12-29

**Authors:** Shuliang Dong, Hongchao Ji, Jian Zhou, Xianzhun Li, Lan Ding, Zhenlong Wang

**Affiliations:** 1 College of Mechanical Engineering, North China University of Science and Technology, No. 21 Bohai Road, Caofeidian Xincheng, Tangshan 063210, China; 2 College of Foreign Languages, North China University of Science and Technology, No. 21 Bohai Road, Caofeidian Xincheng, Tangshan 063210, China; 3 School of Mechatronics Engineering, Harbin Institute of Technology, Harbin 150001, China

**Keywords:** ball sockets, beryllium copper, electrical discharge machining, milling, electrode compensation, gyroscope

## Abstract

Micro-liquid floated gyroscopes are widely used in nuclear submarines, intercontinental missiles, and strategic bombers. The machining accuracy of micro-ball sockets determined the motion accuracy of the rotor. However, it was not easily fabricated by micro-cutting because of the excellent physical and chemical properties of beryllium copper alloy. Here, we presented a linear compensation of tool electrode and a proportional variable thickness method for milling micro-ball sockets in C17200 beryllium copper alloy by micro-electrical discharge machining. The machining parameters were systematically investigated and optimized to achieve high-precision micro-ball sockets when the *k* value was 0.98 and the initial layer thickness was 0.024 mm. Our method provided a new way to fabricate micro-ball sockets in C17200 with high efficiency for micro-liquid floated gyroscopes.

## 1. Introduction

Micro gyroscopes have become a new development direction of gyroscopes owing to their small size, impact resistance, and low cost [1,2]. With the decrease of the structure size of the micro-parts, the motion characteristics of the moving structure are more and more significantly affected by its size effect and surface effect. The micro-components with high precision determine the precision of the gyroscope [3,4]. C17200 beryllium copper alloy, which has excellent thermophysical properties, is the preferred material for supporting parts in the moving parts of a micro-liquid floated gyroscope [5,6]. The micro-ball sockets with high shape precision are necessary to achieve the steady rotation of the rotor. At present, there are two methods, micro-cutting and micro-electrical discharge machining (EDM), for the fabrication of micro-ball sockets in C17200 beryllium copper alloy.

Mohammad et al. used micro-end milling to fabricate microchannels on beryllium copper alloy. Burrs were generated on the side surface of the micro-channels [7]. During the diamond turning of beryllium copper alloy, the tools can cause serious wear [8,9,10]. Cutting forces suddenly increased were observed when the tool comes into contact with hard Be particles [11]. Hung et al. found that the brittle fractured Be particles produce scratches on the machined surface [9]. Sharma et al. simulated the cutting process of beryllium copper material with a diamond tool [11]. The simulation results showed that the interaction between diamond and Be particles could cause plastic deformation of the machined surface and affect the surface quality. Moreover, the hard Be particles interacted with diamond tools to increase tool wear. To sum up, the processing of copper alloy with the micro-cutting method has the problem of large cutting force, surface scratches, severe tool wear, etc.

Compared with micro-cutting, micro-EDM completes the workpiece machining by gasification and melting of materials through the discharge energy between the tool electrode and the workpiece [12,13,14,15]. During the process of machining, there is a discharge gap between the tool electrode and the workpiece. Therefore, there is no macroscopic stress, which reduces the contact stress and the contact residual stress caused by the deformation of parts after machining [16,17]. Micro-EDM milling can realize complex three-dimensional structural processing [18,19]. Furthermore, micro-EDM milling is gradually replacing traditional micro-mold processing as a new process method [20]. However, there are still many problems, such as severe tool electrode wear, low machining accuracy, and poor surface quality. Marrocco et al. studied the effect of pulse type on electrode wear and showed that arc and delayed pulses can effectively reduce electrode wear [21]. Yu et al. proposed a compensation strategy based on uniform electrode wear to solve the severe tool electrode wear [22,23]. Through the reasonable planning of the milling path, the electrode wear only occurred in the axial direction after finishing each layer. The electrode wear length was determined according to the removal volume of the workpiece. The tool electrode was only compensated at the initial point of each layer [23,24]. Nguyen et al. found that the angular radius of the electrode had an important effect on machining accuracy, and the micro-hemispheres and micro-pyramids were completed by electrode online error compensation [25]. Wang et al. successfully machined micro-turbine and complex micro-cavity utilizing micro-EDM milling [26]. Wu et al. proposed to design the complex electrode by micro-wire EDM and thermal diffusion welding. The electrode was used to realize a micro-three-dimensional structure machined by micro-EDM [27,28,29]. The steps on the surfaces of the micro-three-dimensional structures were eliminated by adding lead layers between the electrode layers or by using conical electrodes [30,31]. The micro-three-dimensional structure could be finished by micro-EDM according to designing the shape of the initial tool electrode reasonably [32,33,34]. 

**Figure 1:**
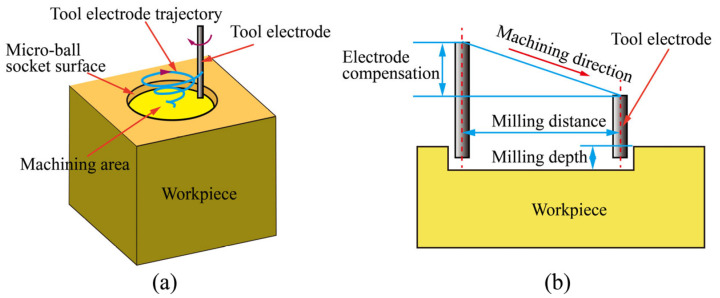
Micro-ball socket milled by micro-EDM. (**a**) Schematic diagram of micro-ball socket milled by micro-EDM (**b**) Schematic diagram of electrode linear compensation.

Herein, we presented a method for the fabrication of the micro-ball sockets in C17200 beryllium copper alloys by micro-EDM milling according to beryllium copper processing characteristics and micro-ball sockets structure requirements. The schematic diagram of micro-ball socket milled by micro-EDM was shown in Figure 1a. The method of linear compensation was proposed to ensure that the fillet angle at the bottom of the tool electrode was kept unchanged (Figure 1b). The corresponding path planning was proposed according to the milling path length, and the influence of layer thickness on the milling accuracy was analyzed. The machining parameters were systematically investigated and optimized. The results showed that our method will be used to fabricate micro-ball sockets in supporting parts of the micro-liquid floated gyroscope.

## 2. Principle of Linear Compensation and Layered Strategy

### 2.1. Linear Compensation of Tool Electrode

During micro-EDM milling, the tool electrode wear mainly occurred in the axial direction of the tool electrode. If effective electrode compensation was not carried out, the accuracy of the three-dimensional structure could be seriously affected [35,36,37]. Uniform wear compensation and fix-length compensation can realize electrode compensation to a certain extent. However, the axial compensation was carried out only at the starting position or the tool electrode milling a certain distance. When the milling path is too long, the compensation method could lead to machining being too depth at the compensation position and insufficient at the end position. Through the analysis of the micro-ball sockets, it was found that the surface of the micro-ball socket was mainly formed by the spark discharge at the end edge of the tool electrode. Therefore, the change in the end radius of the tool electrode could affect the uniformity of the micro-ball sockets. Based on the idea of fix-length compensation and considering the high tool electrode wear of milling beryllium copper alloys, the moving direction of the tool electrode followed a downward diagonal line, as shown in Figure 1b. During milling, the milling depth and the fillet radius of the tool electrical end were constant. 

### 2.2. Layered Strategy of Micro-Ball Sockets

**Figure 2:**
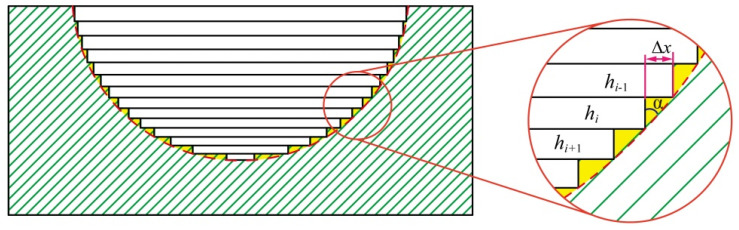
Schematic diagram of changing thickness proportionally.

The machining accuracy of the micro-structure was affected by tool electrode compensation as well as the thickness of each layer, especially when the workpiece contained a curved surface. To improve the effect of the thickness of the layers on machining accuracy, the proportional variable thickness method was adopted, as shown in Figure 2. It can be seen that there is a certain area between layers that cannot be processed, as shown in Figure 2 marked with yellow. The area ∆s is calculated by the equation:

(1) $$\Delta s=0.5\Delta xh_{i}$$

where *h_i_* and ∆*x* are the thickness of *i* layer and one-sided horizontal distance difference between adjacent layers, respectively.

The *h_i_* of the thickness of *i* layer is calculated by the equation:

(2) $$h_{i}=kh_{i-1}\;\left(0<k<1\right)$$

where *h_i_*_–1_ is the thickness of *i* – 1 layer. *k* is the coefficient of equal ratio, 0 < *k <* 1.

The ∆*x* can be calculated by the equation:

(3) $$\Delta x=h_{i}\tan\alpha$$

where α is the angle between the arc contour and the vertical direction.

The area ∆*s* can be expressed as the following equation:

(4) $$\Delta s=0.5h_{i}{}^{2}\tan\alpha$$

In the process of micro-ball sockets milling, α increases with the increase of milling depth. According to Equation (4), ∆*s* increases with the increase of the angle α, when the thickness of every single layer is certain. The machining error in the process of micro-ball sockets also increases, which seriously affects the dimensional accuracy of the micro-ball sockets. Proportional variable thickness method can reduce the thickness gradually with the machining depth, which not only ensures that the number of machining layers will not increase too much but also effectively reduces the machining error at the bottom of the micro-ball sockets.

## 3. Experimental Details

### 3.1. Machine Facilities

**Figure 3:**
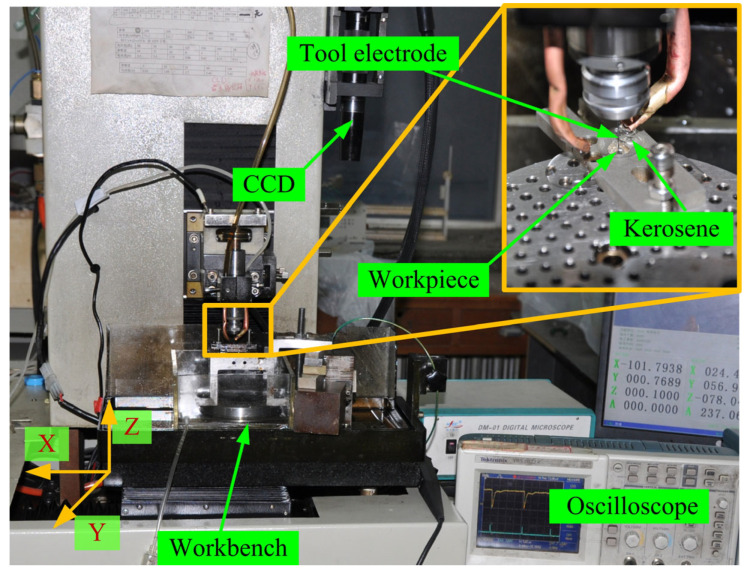
Photograph of experimental set-up.

Experiments were carried out in a micro-EDM machine shown in Figure 3. The positioning and repeatability accuracy for all axes are 200 nm and 100 nm, respectively. The tool electrode was fixed on the *Z*-axis by a rotating joint with a rotating speed up to 4000 rpm. The tool electrode and the workpiece are connected to the negative and positive terminals of the machine, respectively. The polarity of the electrodes is controlled by the machine system. The tool electrode was machined to design diameter by block electrode. The oscilloscope was used to detect the discharge signal during the machining process. The charge coupled device (CCD) was used to measure the diameter and axial wear length of the tool electrode.

### 3.2. Machining Conditions

C17200 beryllium copper alloy (DongGuan Jia Sheng Copper Co., Ltd., Dongguan, China) bar with a diameter of 5 mm cut into a size of 5 mm was used as the workpiece. The impurities of Be, Co, Ni, Si, and Fe in C17200 beryllium copper alloy are 1.90–2.15%, 0.35–0.65%, 0.20–0.25%, <0.15%, and <0.15% by weight, respectively [6]. Cylinder tungsten (DongGuan Gang De Co., Ltd., DongGuan, China) was used as a tool electrode. The diameter of the tool electrode was machined from 500 μm to 150 μm by the block electrode. Kerosene (EDM-3, Mobil, Irving, TX, USA) was used as the dielectric liquid. The detailed machining parameters are presented together in Table 1.

### 3.3. Characterization

The machining time of the micro-ball socket was the average of five measurements under the same conditions. Wire-EDM with a wire electrode diameter of 50 mm was used to cut the micro-ball sockets along the axis of the cylinder of micro-ball sockets. The workpiece was cleaned in alcohol for 15 minutes by ultrasonic, and then the profile was polished by sandpaper (Grit No. 1000). A scanning electron microscope (SEM, Hitachi SU8000) was used to characterize the surface topography of the micro-ball sockets.

## 4. Results and Discussion

### 4.1. Effect of Layered Strategy on Machining Error

**Figure 4:**
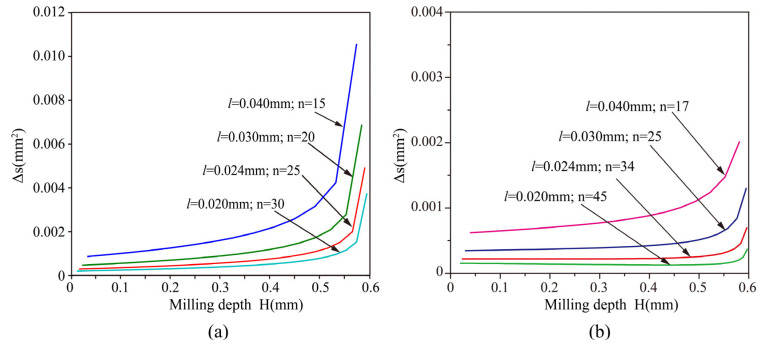
The error of milling with different layered strategies (**a**) constant thickness, (**b**) proportional variable thickness.

The comparison of machining errors under different layered strategies is shown in Figure 4. The initial thickness of the layer is denoted by *l*. The error of milling, when the thickness of each layer is equal to the initial layer, is shown in Figure 4a. It can be seen that the error increases with the increase of the machining depth and decreases with the decrease of the layer thickness. The machining error increases rapidly when the machining depth reaches 0.55 mm. The main reason for this is that the value of α increases at the bottom of the micro-ball socket. Although the machining accuracy can be improved by reducing the thicknesses of the layers, it is impossible to reduce the thickness of the layers indefinitely. 

The required number of layers and machining error under different initial layer thickness, when *k* is 0.98, are shown in Figure 4b. Under the same *k* value, the machining error decreases with the decrease of the initial layer thickness, but the number of layers increases rapidly. By comparing Figure 4a with Figure 4b, it can be seen that the number of layers of the proportional variable thickness method increased slightly, but the machining error is much smaller than that of milling with constant thickness.

### 4.2. Milling Path Generation and Machining Error Simulation

The milling trajectory of the tool electrode could affect the electrode compensation between adjacent layers, especially when the trajectory of the single layer is too long. The tool electrode was first milling from the center. After finishing this layer, the tool electrode machined the next layer along the opposite route and repeated until the micro-ball socket was finished. This trajectory of the tool electrode could effectively reduce the phenomenon that the thickness of the center and edge was not consistent due to electrode wear during single-layer milling. Moreover, this method can effectively reduce the accumulation of errors between different layers caused by electrode wear and the empty travel of the tool electrode.

**Figure 5:**
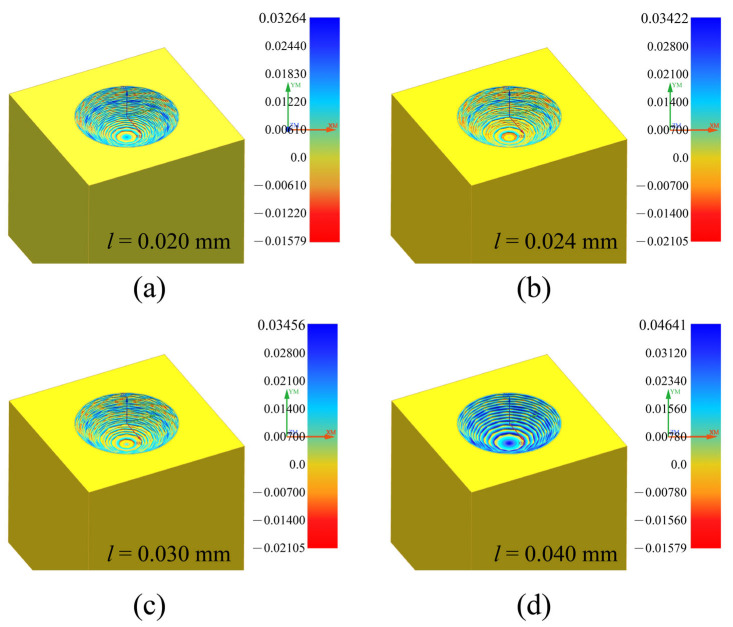
Simulation of the errors with different initial layer thicknesses. (**a**) *l* = 0.020 mm; (**b**) *l* = 0.024 mm; (**c**) *l* = 0.030 mm; (**d**) *l* = 0.040 mm.

Simulation research was conducted on the machining size and design size obtained by the planned machining path. The milling simulation analysis results obtained under different initial layer thicknesses are shown in Figure 5. The simulation results correspond to Figure 4b. It can be seen that the variation of machining error between different layers. When the initial layer thickness is small, the relative machining error is relatively small, as shown in Figure 5a. However, the reduction of the initial layer thickness would increase the milling path, which will be accompanied by an increase in the uncertainty of tool electrode wear. 

### 4.3. Effect of Different Initial Thicknesses on Machining Time

**Figure 6:**
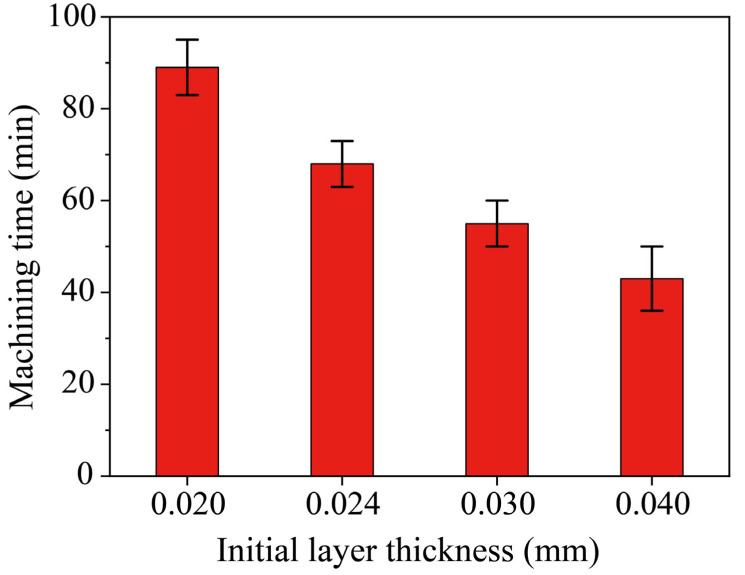
Machining time of ball sockets under different initial layer thicknesses.

Under the same value of *k*, the initial thickness determined the number of layers needed for milling a certain depth of micro-ball sockets. The smaller the initial layer thickness, the greater the number of layers required, as shown in Figure 4b, which leads to a longer path for the tool electrode to scan, and inevitably leads to an increase in the machining time when the same volume of material is removed, as shown in Figure 6. It can be seen that the machining time decreases with the increase of the initial thickness. The reason for this is that the number of layers decreases with the increase of the initial thickness. Although the milling time is not proportional to the number of layers, the milling time will also decrease with the decrease of the milling path. According to Figure 4b, the number of layers of the initial layer thickness of 0.020 mm is 2.64 times the initial layer thickness of 0.040 mm. However, the milling time of layers with an initial layer thickness of 0.020 mm is 2.06 times the initial layer thickness of 0.040 mm, as shown in Figure 6. The relationship between the number of layers and processing is not strictly proportional. The main reason for this is that the removal of single-layer processing will also increase the processing time when the initial layer thickness of 0.040 mm. 

### 4.4. Effect of Different Initial Thicknesses on Shape Precision

**Figure 7:**
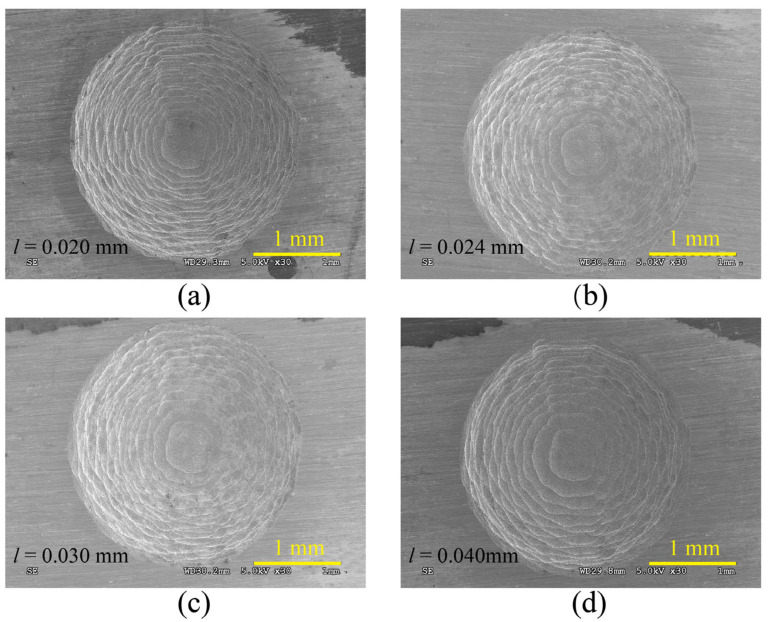
Morphology of ball sockets under different initial layer thicknesses. (**a**) *l* = 0.020 mm; (**b**) *l* = 0.024 mm; (**c**) *l* = 0.030 mm; (**d**) *l* = 0.040 mm.

Figure 7 shows the SEM images of micro-ball sockets with different initial layer thicknesses. It can be seen that the micro-ball sockets structure machined by cylindrical electrode milling could produce milling errors on their surface due to the thickness of layers. The machining error decreases with the decrease in thickness. When milling the same volume of micro-ball sockets, the layers of micro-ball sockets were increased by reducing the initial layer thickness resulting in a longer milling path and increased processing time.

**Figure 8:**
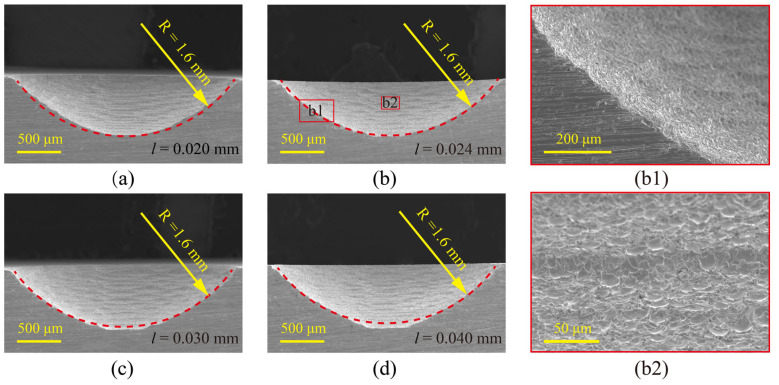
Section and partial enlargement of ball socket under different initial layer thicknesses. (**a**) *l* = 0.020 mm; (**b**) *l* = 0.024 mm; (**c**) *l* = 0.030 mm; (**d**) *l* = 0.040 mm; (**b1**,**b2**) are the local magnification of (**b**).

The micro-ball sockets obtained under different initial layer thicknesses were cut by wire-EDM with a wire electrode diameter of 50 μm. The micro-ball sockets profile structures are shown in Figure 8. The red dashed line in Figure 8 is an arc, which is used to test whether the ball and socket structure profile is an arc surface. The radius of the arc R is 1.6 mm. It can be seen that the machining error on the side of the micro-ball sockets decreases with the increase of the initial layer thickness. However, the error on the bottom will become larger (as shown in Figure 8d). The experimental results show that when the initial layer thickness is 0.024 mm, the profile of the micro-ball socket profile structure has the highest coincidence with the dashed line, as shown in Figure 8b. Figure 8b1,b2 show local enlarged views under this processing condition. The results indicated that the micro-ball sockets manufactured by this method meet the requirements of micro-liquid floated gyroscopes. In addition, the micro-grooves and pit generated by micro-EDM milling can provide a good substrate for anti-drag and lubrication.

## 5. Conclusions

In this paper, high-precision micro-ball sockets were obtained in C17200 beryllium copper alloy by micro-EDM milling with linear compensation of tool electrode and proportional variable thickness method. The machining error, machining time, and shape precision were studied under different initial layer thicknesses. In comparisons of constant thickness, the proportional variable thickness layered strategy can effectively reduce the machining error when the number of layers was slightly increased. The results of the experiments showed that machining time decreased with the increase of the initial thickness. However, the milling time was not proportional to the number of layers. Considering the shape precision of micro-ball sockets, a value of 0.024 mm initial layer thickness can be considered as an ideal initial layer thickness for micro-ball sockets milled by micro-EDM with linear compensation of tool electrode and proportional variable thickness method when the *k* value was 0.98. The results indicated that our method could be used for the fabrication of micro-ball sockets in micro-liquid floated gyroscopes.

## Figures and Tables

**Table 1:** Machining conditions for experiments.

Parameters	Value
Work-piece	*Φ*5 mm × 5 mm of C17200 beryllium copper alloy
Electrode	Tungsten, *Φ*150 μm in diameter
Peak current (A)	0.84
Pulse width/interpulse (μs)	10/10
Revolution of electrode (rpm)	2000
Gap voltage (V)	250
Servo reference voltage (V)	150
Capacitance (1000 pF)	2.2
Initial thickness of the layer *l* (mm)	0.020, 0.024, 0.030, 0.040
Dielectric fluid	Kerosene

## Data Availability

Not applicable.
